# Eudragit Films as Carriers of Lipoic Acid for Transcorneal Permeability

**DOI:** 10.3390/polym15071793

**Published:** 2023-04-05

**Authors:** Karina L. Bierbrauer, Laura R. Comini, Victoria Leonhard, Micaela A. Escobar Manzanelli, Gabriela Castelli, Silvia Farfán, Roxana V. Alasino, Dante M. Beltramo

**Affiliations:** 1 Centro de Excelencia en Productos y Procesos de Córdoba, Gobierno de la Provincia de Córdoba, Pabellón CEPROCOR, Santa María de Punilla, Córdoba CP 5164, Argentina; 2 Consejo Nacional de Investigaciones Científicas y Técnicas, CCT Córdoba, Córdoba CP X5000, Argentina; 3 Facultad de Ciencias Químicas, Universidad Católica de Córdoba, Córdoba CP X5000, Argentina

**Keywords:** Eudragit E100 films, Lipoic Acid, mechanical properties, mucoadhesivity, drug release, corneal permeability

## Abstract

Diabetes mellitus (DM) is a highly prevalent disease affecting almost 10% of the world population; it is characterized by acute and chronic conditions. Diabetic patients have twenty-five times higher risk of going blind and developing cataracts early than the general population. Alpha-lipoic acid (LA) is a highly valuable natural antioxidant for the prevention and treatment of ophthalmic complications, such as diabetic keratopathy and retinopathy. However, its applicability is limited due to its low solubility in water; therefore, suitable systems are required for its formulation. In this work we developed an erodible insert based on Eudragit E100 (*E PO*) and Lipoic Acid (LA) for the delivery of this compound for the preventive treatment of ocular diseases especially in diabetic patients. Film evaluation was carried out by mechanical and thermal properties, mucoadhesivity, drug release, dynamic light scattering and corneal permeability as the concentration of LA increased. It was shown that upon LA release, it forms nanoparticles in combination with *E PO* that favor corneal permeation and LA retention in the cornea. These *E PO*-LA films also resulted non-irritable hence they are promising for their application in the treatment of ocular diseases.

## 1. Introduction

Lipoic acid (LA), also known as α-lipoic acid or thioctic acid, is an organosulfur compound containing a 1,2-dithiolane ring and a carboxylic acid group [1]. LA is a naturally-occurring compound synthesized in small amounts by humans. Endogenously synthesized LA is bound to proteins, functioning as a cofactor for several important mitochondrial enzymes [2]. The potent antioxidant effect of LA has been widely applied to the prevention and treatment of ophthalmic complications [3]. Furthermore, its use in ophthalmic formulations has gained increasing interest following different therapeutic objectives, such as dysfunctions related to glaucoma, prevention of ocular conditions in diabetic patients and treatment of presbyopia and Pterygium [3]. Its application is limited to its solubility due to its hydrophobic nature. 

On the other hand, Eudragit polymers exhibit favorable behavior in the formulation of various active ingredients in a wide range of applications. They are not toxic and offer alternatives of polarity; they show highly versatile mechanical properties and excellent conditions for their use in drug-controlled release [4]. Moreover, some members of this family protect biological products from moisture or light, improve skin penetration and help to seal sensitive actives and increase patient compliance by masking tastes and odors [5]. Eudragit^®^ E100 and Eudragit^®^ E *PO* (dimethylaminoethyl methacrylate copolymer) are cationic copolymers commonly used in dosage forms such as films and tablets to mask flavor [5] or to facilitate drug solubility through formulation which leads to amorphization [6,7]. Eudragit polymers as a carrier system for ophthalmic drug release have been studied for more than a decade. The application of these polymers in ophthalmic drug delivery begins with Eudragit RL100/RS100 in suspension nanoparticle systems, having great potential because they do not present toxicity and, for their high mucoadhesion and controlled drug release [8,9,10,11]. 

An example of the aforementioned is the development of polymeric nanoparticles composed of Eudragit RL 100 and/or Eudragit RS 100 loaded with ibuprofen, amikacin or gatifloxacin/prednisolone as drug carriers to improve drug penetration and bioavailability in the anterior segment of the eye [12]. Another example is the development of dispersions and suspensions of nanoparticles of Eudragit RL100 loaded with fenofibrate for the treatment of diabetic retinopathy and macular degradation, with excellent mucoadhesive properties and sustained drug release [11].

In addition, it is relevant to mention, in relation to the use of Eudragit in ophthalmic administration systems, the development of inserts composed of erythromycin loaded in Eudragit L100 films with significant antibacterial activity [13], as well as the development of nanoparticulate systems albumin–Eudragit S100–melatonin as a promising system for the treatment of ocular neurodegenerative diseases [14], or the development of Eudragit S100 nanoparticles loaded with cyclosporine incorporated into a contact lens matrix to improve the sustained release of the drug [15]. The use of ophthalmic inserts can be advantageous, especially those with synthetic/semi-synthetic polymers offering simple design based on products well suited to ophthalmic use and being easily processed by conventional methods such as slow evaporating extrusion, compression or injection molding [16,17,18].

In addition, drug release occurs by penetration of the tears into the insert which induces its release by formation of a gel layer around the core, which promotes further drug discharge, still controlled by diffusion [19]. To achieve a slow-release rate, we can use a component to completely cover the matrix, or introduce a suitable amount of hydrophobic polymer capable of decreasing the penetration of the tear fluid, thus decreasing drug release without changing the solubility of the insert [20,21]. 

Therefore, in this work, we present a new strategy to increase the availability of LA by incorporating it into Eudragit *E PO* films at different concentrations. The generated films were physicochemically characterized and their mechanical properties, mucoadhesivity, swelling, disintegration and release profiles were evaluated for their potential use in controlled release systems for topical application of drugs. In addition, we also analyzed the corneal permeability and irritability of the films under study in ex vivo pig cornea, to be used as ocular inserts in the preventive treatment of anterior segment diseases.

## 2. Materials and Methods

### 2.1. Materials

Eudragit *E PO* (average molecular weight = 150,000) [5] was supplied by Rhöm Pharma (Darmstadt, Germany). Porcine gastric mucins partially purified from porcine stomach (type III) were purchased from Sigma Chemical Co. (St. Louis, MO, USA). Lipoic acid (LA) was supplied by Todo Droga, Córdoba, Argentina. Polyethylene glycol (PEG400) was purchased from Fluka (Buchs, Switzerland). All other reagents were of analytical grade and used as received. 

### 2.2. Preparation of Eudragit E PO-LA Films

**Figure 1:**
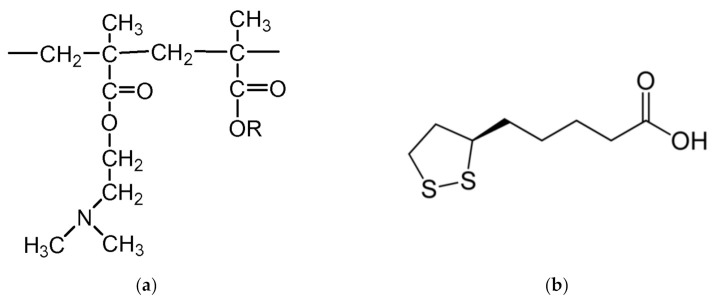
Molecular structure of Eudragit *E PO* (**a**), Lipoic acid (**b**).

Eudragit *E PO* (1 g) (Figure 1) was dissolved in 5 mL of ethanol with 0.06 g PEG400 as a plasticizer (6%); below that concentration no effect was found and at higher concentrations the films became opaque. LA (Figure 1) was then added to the solution at 0.2:1, 0.3:1, 0.4:1 and 0.5:1, LA:E *PO* *w*/*w* ratios (based on dry component weight). The final solution was then casted over PTFE molds (Metalúrgica Gelfo, Córdoba, Argentina with strip shape of 1 × 15 cm to test mechanical properties and over circles of 1.5 and 2.5 cm diameter to evaluate film stability and adhesive properties, respectively. They were left to dry for 1 week at room temperature (25° C) in a closed cabinet at 50% relative humidity. Film thickness ranged from 300 to 350 μm.

### 2.3. Swelling and Disintegration Time Determination

Water uptake was performed to assess the bulk hydrophilicity of films. Swelling was determined by dipping the inserts of 1.5 cm diameter in phosphate buffer saline (PBS, Todo Droga, Córdoba, Argentina) (pH 7.4). Temperature was kept at 37 °C. At regular time periods the films were wiped with lint-free tissue to remove PBS excess, weighed and returned to the same container. All measurements were performed in triplicate. The maximum water uptake (% Swelling) of the films was determined according to the following expression (Equation (1)):

(1) $$\%\;Swelling=\left[\frac{Ws-Wd}{Wd}\right]\times \;100$$

where *W_s_* is the weight of swollen film at different times and *W_d_* is the weight of dry film.

These same samples were left submerged until total disintegration to evaluate their time stability.

### 2.4. Evaluation of the Mechanical Properties of Films

The mechanical properties of the inserts (tensile strength, elongation to break and elastic modulus) were determined using a TA-XT2i texturometer (Stable Micro Systems Ltd., Surrey, Godalming, UK) in tensile strength mode. In these assays the films used were rectangular strips. The initial grip separation was set as 30 mm, and the crosshead speed was maintained at 30 mm min^−1^, without preload. Mechanical properties were analyzed from stress versus strain plot. The mechanical properties for each type of insert were carried out in triplicate and average values were reported.

### 2.5. Evaluation of the Mucoadhesivity

Film mucoadhesivity was measured as the force required by detaching an insert from a reconstituted mucin layer according to the method reported by Rossi et al. [20]. In vitro mucoadhesion was tested using the above mentioned texturometer TA-XT2i (Stable Micro Systems Ltd., Surrey, Godalming, UK)consisting of a lower and an upper plate. The lower plate was fixed, while the upper plate (25 mm diameter) was connected to a movable load cell, measuring force of detachment. For mucoadhesion testing, reconstituted mucin was spread on the lower plate and the inserts (25 mm diameter) were fixed to the upper plate using cyano acrylate adhesive. The upper plate was then lowered. A preload of 0.5 N was applied for 60 s and the probe was then raised at a constant speed of 0.1 mm s^−1^ until the insert was completely detached from the mucin layer. This detachment point represents the adhesive bond strength between these surfaces. The force required for complete detachment of the insert from the mucin layer was determined by computer-integrated data acquisition Texture Analysis software. The integration of the areas of force vs. distance curves which represent the adhesion work were expressed in units of centiNewton.mm (cN.mm). The text was performed in triplicate for each type of insert and mean values of adhesion work are reported.

### 2.6. Differential Scanning Calorimetry Measurements

For these measurements, a Differential Scanning Calorimetry (DSC) 800 Perkin Elmer equipment (Perkin Elmer, Buenos Aires, Argentina) was used, in which a reference sample and the sample of interest were placed. The sweep temperature started at 25 °C up to 110 °C, at a heating rate of 10 °C min^−1^, in N_2_ stream.

The objective of this technique was to measure the heat capacity of a system when undergoing a transition induced by a thermal change by measuring the energy transferred in the form of heat flux between two systems at a given time. From the thermograms, the following parameters were obtained: enthalpy values that represent the total heat corresponding to the transformation produced in the sample; the area under the curve which is equivalent to the increase in enthalpy of the process; and the maximum peak of the sample defined as the transition temperature of the material.

### 2.7. Drug Release from Eudragit E PO Films

Casted films of 1.5 cm diameter were placed in tubes with one end open where semi-permeable SpectraPor dialysis membranes (OmniLab SRL, Buenos Aires, Argentina) with an MW cut-off of 12–14 kDa were set up (donor chamber). These tubes with 2 mL of PBS pH 7.4 were immersed in another recipient containing 40 mL of the same PBS (receptor chamber). The recipients were placed in a shaker at 150 rpm and 37 °C. At predetermined time intervals, the whole 40 mL volume was replaced by fresh PBS, thus, perfect sink conditions were maintained all the time.

The withdrawn samples were assayed spectrophotometrically for drug content using a UV-visible spectrophotometer (Perkin Elmer, USA) at 334 nm. Finally, the concentration of LA was determined from a previously created calibration curve. The LA profile of each film was expressed as accumulative percentage of drug vs. time. To obtain LA solubility, an excess of LA (10 mg) was weighed in Eppendorf tubes (Axygen, Buenos Aires, Argentina) (in triplicate) and 1 mL of PBS pH 7.4 was added. The tubes were kept under stirring for 24 h at 600 rpm and 25 °C. Then they were centrifuged at 14.000 rpm and 25 °C for 15 min, to separate non-solubilized LA. Finally, the supernatants were extracted to subsequently quantify them by UV–Vis spectroscopy previous LA calibration curve at 334 nm (UV/VIS spectrophotometer Perkin Elmer, Buenos Aires, Argentina). The LA solubility obtained under these conditions was (1.99 ± 0.12) mg mL^−1^. According to previous work [3] LA solubility in carbonate buffer pH 7.2 resulted in 1.22 mg/mL close to our result.

Profiles were evaluated using the Ritger–Peppas model [22] for early release time. This model relates the release of the drug with the elapsed time raised to an exponent *n*, employing data release below 60% according to the following equation (Equation (2)):

(2) $$\frac{M_{t}}{M_{\infty }}=kt^{n}$$

where *M_t_*/*M*_∞_ is the percentage of drug released at time *t*, *k* is the constant release, *t* is the elapsed time and *n* is the exponent of liberation.

### 2.8. Particle Size and Zeta Potential

The average particle size, polydispersity and zeta potential generated during the release of LA contained in the *E PO* films were determined. For this, the *E PO*-PEG400-LA films at different LA concentrations were submerged in 10 mL of PBS (pH 7.4) at 37 °C, and 1 mL aliquots were taken at different times, replacing that volume with new PBS. These values were also measured for PBS alone as well as *E PO*-PEG400 and LA in PBS. To carry out these experiments a dynamic light scattering (DLS) Malvern Instrument NANO ZS (CAS Instrumental, Buenos Aires, Argentina) was used at a fixed scattering angle of 173°. The chemical characterization of the nanoparticles obtained was carried out by FTIR spectroscopy. Procedure details and spectra are shown in the Supplementary Material (Figure S1).

### 2.9. Corneal Irritability Evaluation

The corneal irritability test was performed using an adapted method from the recently revised Test N° 437 to assess ocular tolerability [23]. In our case, we use ex vivo pig eyes as a model. Pig eyes were previously incubated in saline solution at 37 °C for 10 min. The films to be tested were placed on the cornea surface and left to act for 5 h with a continuous instillation of physiological solution to simulate blinking. Subsequently, the remaining film was removed and the cornea surface was rinsed with physiological solution. At the same time, a positive control was performed by placing a piece of paper soaked in NaOH 1 M in another eye, which was left to act for 30 s and then rinsed with physiological solution. 

The degree of corneal damage was determined visually by the extent of the opacity and evaluated using sodium fluorescein (2% *w*/*v*) staining method under cobalt blue light (465–490 nm) [23].

### 2.10. Corneal Permeability

Corneal permeability was performed using ex vivo pig corneas, which were placed between the donor chamber (1 mL) and the receptor chamber (7.5 mL) of a Franz cell. The film to be evaluated was placed on the cornea. The two chambers were filled with PBS and the cell was thermostatted at 37 °C. Perfect sink conditions were maintained throughout the experiment. Aliquots of 0.5 mL were taken from the receptor chamber at different times and the same volume was replaced by PBS. 

At the end of the experiment, the excess film was removed from the cornea, and the LA was extracted in 2 mL with ethanol for subsequent analysis. The qualitative and quantitative analysis of LA in each aliquot (taken at different times) was carried out by HPLC using a ultra high perfomance chromatograph UHPLC model Nexera XR, Jenck, Buenos Aires Argentina, equipped with a PDA detector model SPD-M30A and a column Shimpack GIST C_18_ (100 × 2.1 mm I.D., particle size 2 μm) and a guard column Gist HP (G) C_18_ guard (10 × 2.1 mm I.D., particle size 2 μm) supplied by Shimadzu, Jenck, Buenos Aires Argentina. The mobile phase was a mixture of methanol and water 70/30, the pH was adjusted to 3.0 with the help of phosphoric acid, the flow rate was set at 0.1 mL min^−1^, and the injection volume was 10 μL. Chromatography was performed at 40 °C, and detection was set up at a wavelength of 332 nm. The identification of LA in the different samples was carried out by comparing the HPLC retention time (t_R_) of the corresponding standard. The calibration method with an external standard was used to quantiy LA in the different samples. The calibration curve of the corresponding standard was drawn up linear range 0.5–200 μg mL^−1^ Y = 31,316X + 2200 (r^2^ = 0.99999, *p* < 0.0001), where Y is the peak area given by data processor and X is the amount of LA expressed as μg mL^−1^.

### 2.11. Statistical Analysis

Data analysis was carried out using one-way analysis of variance (ANOVA). Data were expressed as mean ± standard deviation (SD). Significance was accepted with *p* < 0.05.

## 3. Results and Discussion

### 3.1. Effect of LA on the Mechanical Properties of Eudragit E PO Films

**Figure 2:**
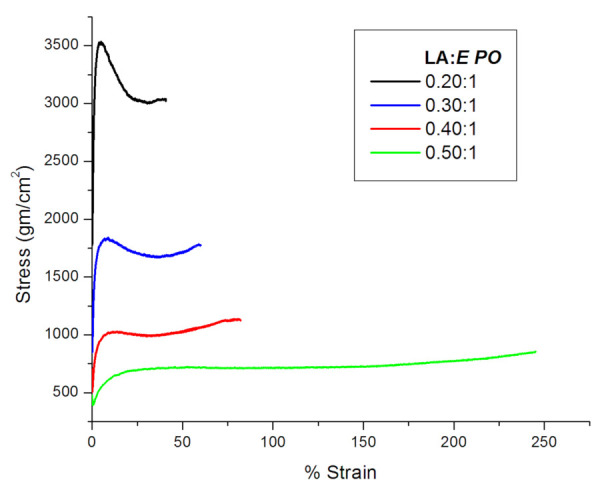
Effect of increasing LA concentrations in the mechanical properties of *E PO*-LA films.

We evaluated the effect of LA on the mechanical properties of *E PO* films. PEG400 was used fixed at an optimal concentration of 6% (*w*/*w*) in all samples. According to this, Figure 2 shows the profiles of stress vs. strain for Eudragit *E PO*-PEG400 6% with increasing content of LA.

As can be seen, there is a decrease in the stress and a consequent increase in the strain as LA concentration grows up. Therefore, increasing amounts of LA produce a plasticizing effect in *E PO* films. Table 1 summarizes the results of tensile strength and Young’s modulus, both obtained from Figure 2 curves. This table shows that both Young’s modulus and tensile strength decrease at high LA:*E PO* ratio (0.4:1 and 0.5:1). It is well known that plasticizers reduce the forces of attraction between polymeric chains, thus increasing their mobility. This behavior is what decreases both tensile strength and Young’s modulus [4].

### 3.2. Film Swelling and Stability

**Figure 3:**
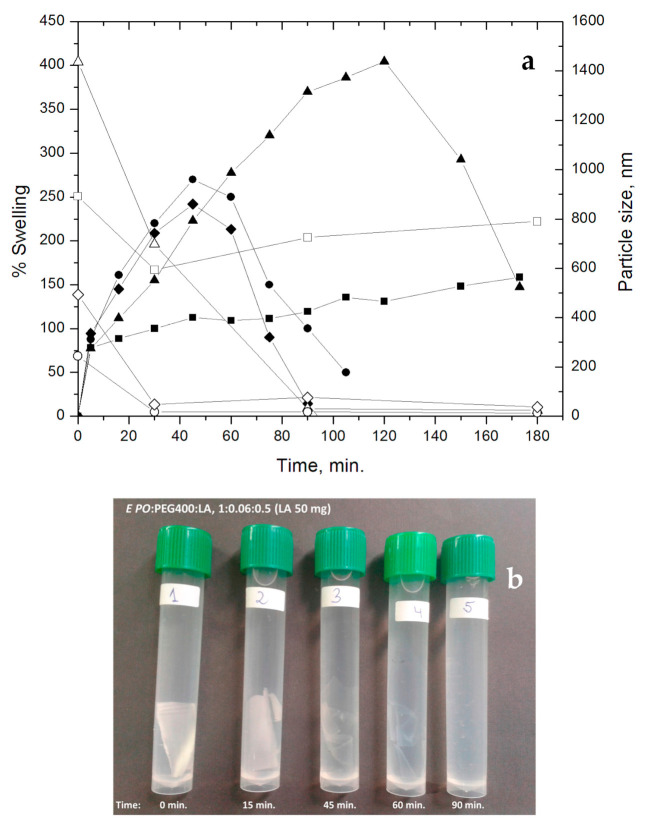
(**a**) Swelling (closed symbols) and particle size (open symbols) vs time of *E PO* films containing increasing quantities of LA (mg): (■;☐) 20, (▲;Δ) 30, (●;◯) 40, (♦;⬦) 50; (**b**) Film disintegration for LA (50 mg), in PBS at 37 °C.

We also evaluated the effect of LA on other physical film properties such as swelling and stability. Both properties were assayed in the same experiment under physiological conditions (PBS and 37 °C), until complete dissolution of the films (Table 1 and Figure 3). For this purpose, *E PO* films with different LA:*E PO* ratios were tested, that is 0.2:1 (LA 20 mg); 0.3:1 (LA 30 mg); 0.4:1 (LA 40 mg); 0.5:1 (LA 50 mg). These results show that an increase of LA raises swellability and reduces the disintegration time of the films, from 15 days (without LA) to 1.5 h (at 0.5:1, LA (50 mg):*E PO*), demonstrating that the presence of LA modulates not only the tensile strength but also the swelling of the film and stability.

Figure 3 shows that, at shorter times, the films swell rapidly, especially those with the highest proportion of LA:*E PO* (0.4:1, 0.5:1). Then, in the case of the film LA:*E PO* 0.2:1, swelling increases gradually, the sample showing the lowest swelling index (75–130%). In the case of the film LA:*E PO* 0.3:1, a higher degree of swelling is observed, reaching a maximum at 2 h (400%) followed by a decrease due to film disintegration. In the case of films 0.4:1 and 0.5:1, the disintegration process occurs earlier (1–1.5 h) since at these ratios the films become more unstable. This property coincides with the greater flexibility observed for these films according to the mechanical properties already shown.

The explanation for this behavior is that LA molecules interpose between *E PO* polymer chains, thus decreasing the interaction between them. In this way, a free volume is generated which can be occupied by the entry of PBS, facilitating swelling and subsequent film degradation. This phenomenon is increased with the initial concentration of LA. In Figure 3b can be observed how this process occurs macroscopically.

### 3.3. Mucoadhesivity

Mucoadhesivity is defined as the force required to separate films from generated mucin layer. This property is very important for topical application since a good mucoadhesivity favors residence time and, in some cases, permeability. In this respect, polymers such as poly (acrylates) have good mucoadhesive properties as they form joints of bridge-type hydrogen or ionic interactions with mucus. A previous work [24] describes interaction with mucus tissues which can be hydrophobic, electrostatic or through disulfide bridges. Therefore, the bases for good adhesive properties are interaction and interpenetration between polymer chains and mucus, and these could be hydrophobic or hydrophilic in nature. In addition, this phenomenon is closely related to the mobility of polymer chains. A compromise between chemical interaction and mobility is needed.

Table 1 shows the adhesion work expressed as cN.mm of *E PO* films at different LA proportions. It can be seen that the variation of LA significantly affects mucoadhesivity as the concentration of LA increases.

Note that LA contains an acidic group and a hydrophobic domain where the S-S bridges are located. Therefore, as *E PO*-LA films are more flexible with increasing amounts of LA, chain mobility increases, facilitating interpenetration and interaction with mucin. 

### 3.4. DSC

**Figure 4:**
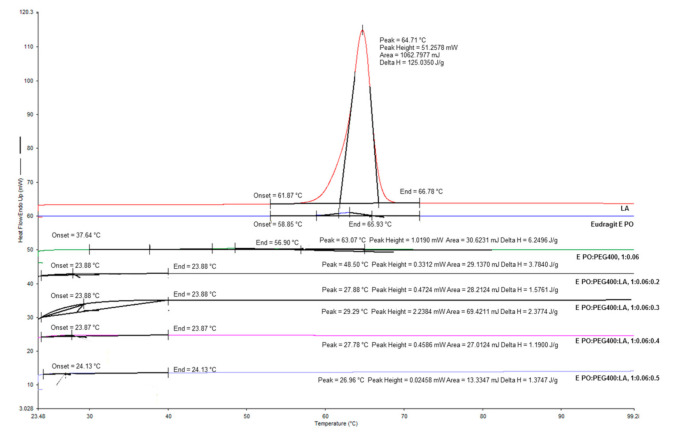
DSC thermograms for Eudragit *E PO*, LA and *E PO* films at different LA concentrations.

Thermograms of *E PO* films with increasing amounts of LA were examined (Table 2 and Figure 4). The films were compared to pure *E PO* and pure LA. The values of enthalpy of fusion (ΔH) showed a reduction from 5.86 J.g^−1^ for *E PO* to 4.45 J.g^−1^ for the film composed of *E PO*-PEG400, which indicates that the addition of PEG400 reduces *E PO* crystallinity. Increasing amounts of LA further decreased ΔH values. It is well-known that plasticizers reduce the forces of attraction between polymeric chains, thus increasing their mobility. This is shown in the decrease of the melting temperature and in the ΔH of the transition [4]. The crystalline region in a polymer matrix hinders the permeation of solutes, hence a reduction in crystallinity significantly improves the release of the compounds inside them [25]. Therefore, LA is acting as a plasticizer for *E PO* films.

### 3.5. LA Release

**Figure 5:**
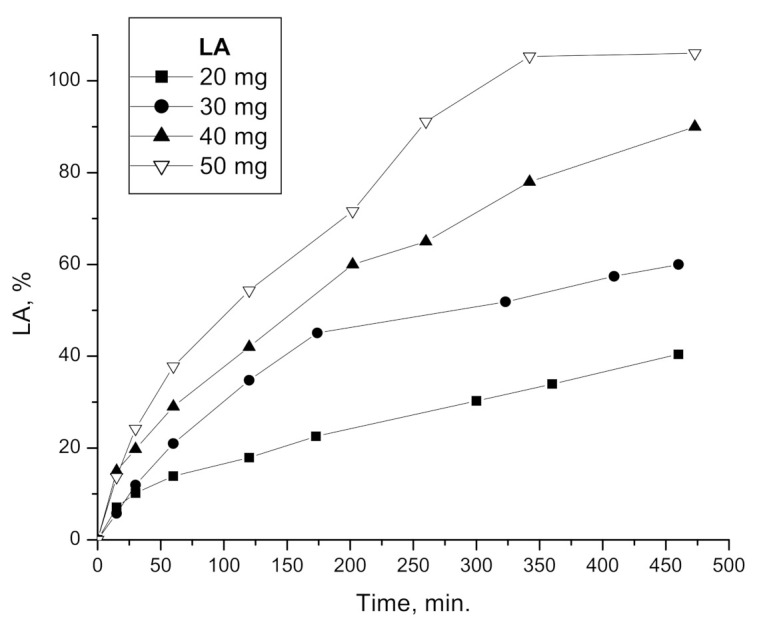
In vitro release of LA from matrix films composed of *E PO* at different LA initial quantities.

Figure 5 shows the release curves of *E PO* films loaded with different ratios of LA:*E PO*. It can be observed that LA release rate increases with the initial concentration of LA. These results agree with the trend observed in swellability assays, since higher LA concentration increases the degree of swelling, therefore LA is available to diffuse at shorter times.

Table 3 shows the release parameters obtained from fitting release results of Figure 5, for the first 6 h drug delivery, applying Equation (2).

In all cases, the diffusional exponent indicates an anomalous diffusion case, except for LA 20 mg. Anomalous release behavior is intermediate between Fickian and Case-II. This is reflected by the fact that anomalous behavior is defined by values of *n* between 0.50 and 1 [22]. 

**Figure 6:**
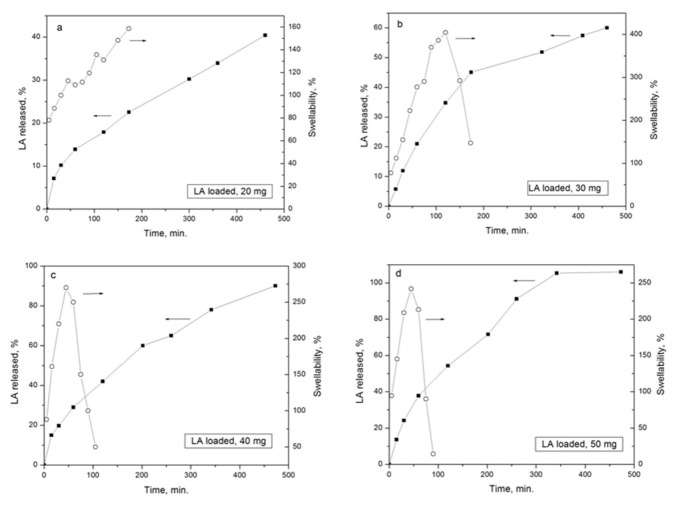
Comparison between release profiles (closed symbols, **left arrows**) and swelling (open symbols, **right arrows**) curves of *E PO* films with different initial amounts of LA.

Figure 6 shows release profiles compared with swelling profiles for each film loaded with increasing initial amounts of LA. The swelling rate of the film with LA 20 mg (Figure 6a) is lower than its release rate, therefore the release behavior is closer to the diffusion-controlled case (*n* = 0.50). On the other hand, swelling rate of films with 30, 40 and 50 mg of LA (Figure 6b–d) is greater, and the relaxation process becomes more important. However, in these cases, both processes are taking place simultaneously, therefore, this is an anomalous case, whose release behavior is intermediate between Fickian and Case II (*n* between 0.50 and 1). 

The release process in our system is complex, since swelling, drug diffusion and film degradation occur in parallel [26]. Some parameters such as constant release, *k*, from Equation (2) does not make physical sense in this case [22]. 

### 3.6. Particle Size and Zeta Potential

Table 4 shows the results of the average particle size, polydispersity and zeta potential generated during the release of LA contained in the *E PO* films. Measurements of PBS alone as well as *E PO*-PEG400 and LA, both in PBS, are shown in the same table. It can be observed that in the *E PO*-PEG400 films loaded with different concentrations of LA, the size of the released particles decreases as a function of time, reaching nanometric values, referring to structures with a length scale applicable to nanotechnology, generally established between 1 and 100 nanometers (nm) [27]. This nanometric size of the released particles is observed at 24 h in films loaded with LA 20 mg; in films loaded with 30, 40 and 50 mg, this phenomenon is observed at shorter times: 1.5 (for LA 30 mg), and 0.5 h (for LA 40 and 50 mg). This behavior is related to the Z potential charge change, mainly for LA 30, 40 and 50 mg since, when particle sizes become nanometric, the Z potential becomes positive. It can also be seen that PBS only has a particle size of 19.4 nm with a negative Z potential. When LA solid in PBS (LA/PBS solubility 1.99 mg/mL) is analyzed, the average particle size varied from 110 to 745 nm, also with a negative Z potential value. 

On the other hand, in the case of the *E PO*-PEG400 film without LA, the formation of larger particle sizes is observed, probably due to the release of PEG400 from the *E PO*-PEG400 matrix.

These results suggest that, depending on the LA loaded, there is an in-situ release of particles with nanometric structure between LA and *E PO* polymeric chains, where probably LA is being retained within the *E PO* film exposing the positive charges of the latter, enabling a greater interaction of this system with the body objectives. Figure 3 shows the correlation found between particles size and swellability. It can be seen that as films swell and disintegrate, particle size becomes smaller. Due to the complicated anatomical and physical properties of the ocular structure, targeted drug delivery to these tissues remains a challenge for scientists. Currently, one of the non-invasive and locally administered promising strategies to improve the penetration of ocular barriers is nanotechnology/nanomedicine for the development of nanotherapeutics that are characterized not only by high corneal permeability but also by sustained bioactive delivery. In our study, although the system consists of a device (film) with a homogeneous composition between Eudragit *E PO* and LA, the nanoparticles detected by DLS are generated in situ, during swelling, degradation and release process. These results make this novel formulation an advantageous system, since it allows the active principle to have a greater penetration capacity due to nanometric size of the released structure and efficient cellular internalization, as occurs in other similar systems previously reported in the bibliography [28,29].

### 3.7. Corneal Permeability

**Figure 7:**
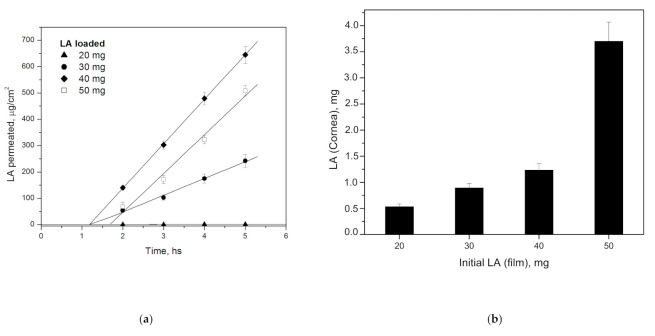
(**a**) Corneal permeability of *E PO*-PEG400 loaded with different initial amounts of LA at different times. Linear regression with *R*^2^ of 0.76 for LA 20 mg and 0.99 for LA 30, 40, 50 mg, respectively. (**b**) LA retained in cornea at different initial LA concentrations after 5 h.

Figure 7a shows the amount of LA per unit area that permeated from *E PO*-PEG400 films through cornea as a function of time. In this graph, a linear relationship of LA permeation is observed in all cases. From the slopes, can be obtained the steady state flux (*J*) and from the x-intercept, the lag time (*t_lag_*). In the film with LA 20 mg, permeability is very low. Then, LA 30 mg presents a J of 63.7 mg/cm^2^.h and *t_lag_* of 1.17 h; LA 40 mg shows a J of 169.0 mg/cm^2^.h with *t_lag_* of 1.18 h, while LA 50 mg shows a J of 147.4 mg/cm^2^.h with *t_lag_* of 1.69 h. It can be seen that the flux is dependent on the initial LA concentration. 

As seen, particle size becomes smaller at shorter times when LA film initial concentration is higher (Table 4), thus, this property could ease LA cornea permeability. A fact appears with the LA 40 mg film, whose profile shows a greater slope and lower *t_lag_* than that of LA 50 mg film. This may be due to the fact that in the LA 50 mg film, a saturation of corneal ducts would cause permeability to decrease in relation to LA 40 mg film. This is also consistent with the greater retention in cornea found in LA 50 mg compared to the other concentrations, seen in Figure 7b.

Finally, based on the results obtained, good corneal permeability and corneal retention can be obtained from 30 mg of LA loaded. The differences are fundamentally attributed to the initial LA concentration, which produces similar effects from this concentration. We have been able to demonstrate that permeability can be controlled and adapted to the therapeutic dose needed.

There are two main methods for ocular treatment, one is the application of active principles in the posterior eye segment [30], in which a more or less complicated intervention is required, generally through the application of an injection with all the disadvantages that it implies. Another method is topical application in the anterior segment of the eye. This method is more benign but carries the difficulty that the cornea offers a significant barrier [31]. Previous studies have developed systems in this direction, especially in the use of polymeric nanoparticles [29,32,33] for the treatment of different ocular pathologies. In our case, and taking these previous works as a reference, we can say that the composition and characteristics of our insert favor corneal permeability due to several factors. In the first place, the degradative effect that LA confers on the polymeric matrix. In the second place, due to this degradation effect, *E PO*-LA nanoparticles of small size are formed which facilitate corneal penetration of active principle. 

### 3.8. Corneal Irritability

The fluorescein staining test is simple and quick to predict ocular irritability, thus avoiding the use of animal experiments, as suggested by The European Union Reference Laboratory for alternatives to animal testing [34].

**Figure 8:**
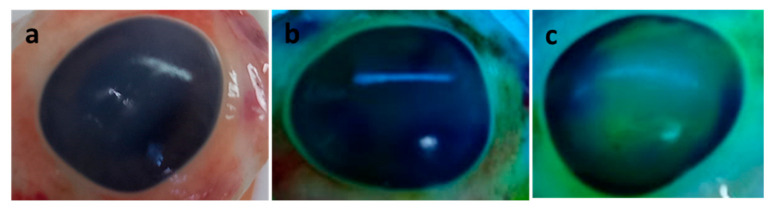
Evaluation of corneal irritability, (**a**) fresh eye before the test; (**b**) 5 h after film in contact with the ocular surface: *E PO*:PEG400:LA, 1:0.06:0.5 (LA 50 mg); (**c**) positive control, NaOH 1M.

Figure 8 shows the results of the corneal irritability test using the sodium fluorescein (2% *w*/*v*) staining method under cobalt blue light (465–490 nm). It can be seen that *E PO*:PEG400:LA, 1:0.06:0.5 film, with LA at the highest concentration, produces no irritability (grade 1) compared to positive control eye (NaOH 1M) (grade 4), according to grade scale from Efron et.al. [35]. Therefore, *E PO*-PEG400-LA films are suitable for ocular application. 

## 4. Conclusions

In this work we studied the effect of LA added to films composed of *E PO*-PEG400. The addition of LA improved the mechanical and adhesion properties of the films of *E PO*-PEG400. In addition, the behavior of the films changed according to the concentration of LA, showing different features related to stability and release. LA produces a plasticizing effect, since films with increasing concentrations of this component are more flexible, improving mucoadhesivity, and accelerating film disintegration times, diffusion and corneal permeability. Therefore, release is controlled by swelling of the films, being favored with increasing amounts of LA. Added to all these advantageous characteristics, we can demonstrate that *E PO*-PEG400-LA films are not irritating and that we can control LA release adjusting its dose to the case to be treated. 

## Figures and Tables

**Table 1:** Effect of LA concentration on the mechanical, mucoadhesive properties and disintegration time of *E PO* films *.

LA:*E PO* *w*/*w* Ratio	Tensile Strength ± SD (N/cm^2^)	Young’s Modulus ± SD (N/cm^2^)	Adhesion Work ± SD (cN.mm)	Film Desintegration Time *
0.0:1	-	-	2.69 ± 0.14	>15 d
0.2:1	11.62 ± 1.27	3.67 ± 0.23	5.73 ± 0.31	24 h
0.3:1	6.00 ± 0.54	1.84 ± 0.10	10.47 ± 0.62	3 h
0.4:1	3.41 ± 0.27	0.73 ± 0.03	13.15 ± 0.70	2 h
0.5:1	2.48 ± 0.19	0.42 ± 0.02	14.24 ± 0.74	1.5 h

* Films dipped in PBS at 37 °C; d: days; h: hours.

**Table 2:** Entalphy values (ΔH) for Eudragit *E PO,* LA and *E PO* films at different LA concentrations.

Sample	Transition Temperature (°C)	ΔH (J g^−1^)
LA	64.7	125.03
Eudragit *E PO*	63.1	6.25
*E PO*:PEG400, 1:0.06	48.5	3.78
*E PO*:PEG400:LA, 1:0.06:0.2	27.9	1.58
*E PO*:PEG400:LA, 1:0.06:0.3	29.3	2.38
*E PO*:PEG400:LA, 1:0.06:0.4	27.8	1.19
*E PO*:PEG400:LA, 1:0.06:0.5	26.9	1.37

**Table 3:** Effect of drug concentration on diffusional coefficient, n, from EPO-PEG400-LA films.

LA Loaded, (mg)	*n*	*r^2^*
20	0.493 ± 0.015	0.997
30	0.828 ± 0.040	0.996
40	0.537 ± 0.012	0.998
50	0.621 ± 0.018	0.997

**Table 4:** Particle size and zeta potential of the nanoparticles generated by LA liberation from *E PO* films in PBS at 37 °C.

Sample *E PO*:PEG400:LA	Time (h)	Particle Size (nm)	Polydispersion Index	Zeta Potential (mV)
1:0.06:0.2	0	891.5	0.74	−6.9
	0.5	593.6	0.50	−3.8
	1.5	725.0	0.62	−0.1
	3	789.6	0.77	+0.8
	6	661.3	0.63	+10.9
	24	78.0	0.32	+15.3
1:0.06:0.3	0	1436.0	0.755	−8.2
	0.5	697.5	0.483	−2.8
	1.5	31.4	0.317	+7.9
	3	23.5	0.361	+13.1
	6	20.9	0.308	+11.9
	24	20.1	0.381	+12.6
1:0.06:0.4	0	244.2	0.40	−4.2
	0.5	17.2	0.45	+11.4
	1.5	17.4	0.42	+6.9
	3	13.1	0.29	+13.9
	6	24.4	0.34	+13.7
	24	12.3	0.28	+14.5
1:0.06:0.5	0	494	0.51	−15.7
	0.5	47.5	0.36	+16.6
	1.5	76.3	0.34	+15.3
	3	37.8	0.27	+16.3
	6	34.2	0.25	+17.2
	24	55.5	0.34	+18.6
*E PO-PEG400*/PBS 1:0.06	0	1230.0	0.83	−9.3
	3	3261.0	0.86	−10.3
	6	4271.0	0.53	−10.7
	24	2376.0	0.83	−15.2
LA (50 mg)/PBS	0	109.8	0.35	−21.8
	3	112.8	0.62	−21.3
	6	744.9	0.55	−17.6
	24	309.7	0.54	−21.3
PBS	-	19.4	0.52	−16.7

## Data Availability

Not applicable.

## Supplementary Materials

The following are available online at https://www.mdpi.com/article/10.3390/polym15071793/s1, Figure S1: FITR spectra of E PO, LA and E PO-PEG400-LA nanoparticles generated from E PO-PEG400-LA (50 mg) film incubated in PBS at 37 °C for 24 h.

## References

[1] Burt Berkson M.D. (1998). The Alpha Lipoic Acid Breakthrough.

[2] Biewenga G.P., Haenen G., Bast A., Fuchs J., Packer L., Zimmer G. (1997). Lipoic Acid in Health and Disease. Book an Overview of Lipoate Chemistry.

[3] Alvarez-Rivera F., Fernandez-Villanueva D., Concheiro A., Alvarez-Lorenzo C. (2016). α-Lipoic Acid in Soluplus® Polymeric Nanomicelles for Ocular Treatment of Diabetes-Associated Corneal Diseases. J. Pharm. Sci..

[4] Elgindy N., Samy W. (2009). Evaluation of the mechanical properties and drug release of cross-linked Eudragit films containing metronidazole. Int. J. Pharm..

[5] Eudragit Acrylic Polymers for Pharmaceutical Applications. Röhm Deguss-Hüls Group. Pharma Polymers. https://www.pharmaceuticalonline.com/doc/rohm-america-0003.

[6] Priemel P.A., Laitinen R., Grohganz H., Rades T., Strachan C.J. (2013). In situ amorphisation of indomethacin with Eudragit® E during dissolution. Eur. J. Pharm. Biopharm..

[7] Doreth M., Löbmann K., Grohganz H., Holm R., Lopez de Diego H., Rades T., Priemel P.A. (2016). Glass solution formation in water—In situ amorphization of naproxen and ibuprofen with Eudragit® E PO. J. Drug Deliv. Sci. Technol..

[8] Pignatello R., Bucolo C., Ferrara P., Maltese A., Puleo A., Puglisi G. (2002). Eudragit RS100 nanosuspensions for the ophthalmic controlled delivery of ibuprofen. Eur. J. Pharm. Sci..

[9] Safwat S.M., Al-Kassas R.S. (2002). Evaluation of gentamicin-Eudragit microspheres as ophthalmic delivery systems in inflamed rabbit’s eyes. STP Pharma Sci..

[10] Glaessi B., Siepmann F., Tucker I., Rades T., Siepmann J. (2010). Deeper insight into the drug release mechanisms in Eudragit RL-based delivery systems. Int. J. Pharm..

[11] Khin S.Y., Soe H.M.S.H., Chansriniyom C., Pornputtapong N., Asasutjarit R., Loftsson T., Jansook P. (2022). Development of Fenofibrate/Randomly Methylated β-Cyclodextrin-Loaded Eudragit® RL 100 Nanoparticles for Ocular Delivery. Molecules.

[12] Janagam D.R., Wu L., Lowe T.L. (2017). Nanoparticles for drug delivery to the anterior segment of the eye. Adv. Drug Deliv. Rev..

[13] Mirzaeei S., Taghe S., Alany R.G., Nokhodchi A. (2022). Eudragit® L100/Polyvinyl Alcohol Nanoparticles Impregnated Mucoadhesive Films as Ocular Inserts for Controlled Delivery of Erythromycin: Development, Characterization and In Vivo Evaluation. Biomedicines.

[14] Martinez S.M., Inda A., Garcia A.M., Bermúdez J.M., Gonzo E.E., Herrero-Vanrell R., Luna J.D., Allemandi D.A., Quinteros D.A. (2022). Development of melatonin-loaded, human-serum-albumin nanoparticles formulations using different methods of preparation for ophthalmic administration. Int. J. Pharm..

[15] Nguyen D.D., Lai J.Y. (2020). Advancing the stimuli response of polymer-based drug delivery systems for ocular disease treatment. Polym. Chem..

[16] Rathore K.S., Nema R.K. (2009). Review on ocular inserts. Int. J. Pharmtech. Res..

[17] Chang J.N., Kulkarni V.S. (2010). Recent advances in ophthalmic drug delivery. Handbook of Non-Invasive Drug Delivery Systems.

[18] Jain D., Carvalho E., Banerjee E. (2010). Biodegradable hybrid polymeric membranes for ocular drug delivery. Acta Biomater..

[19] Wang R., Lu D., Wang H., Zou H., Bai T., Feng C., Lin Q. (2021). “Kill-release” antibacterial polysaccharides multilayer coating based therapeutic contact lens for effective bacterial keratitis treatment. RSC Adv..

[20] Rossi S., Bonferoni M.C., Ferrari F., Bertoni M., Caramella C. (1996). Characterization of mucin interaction with three viscosity grades of sodium carboxymethylcellulose. Comparison between rheological and tensile testing. Eur. J. Pharm. Sci..

[21] Moiseev R.V., Steele F., Khutoryanskiy V.V. (2022). Polyaphron formulations stabilised with different water-soluble polymers for ocular drug delivery. Pharmaceutics.

[22] Ritger P.L., Peppas N.A. (1987). A simple equation for description of solute release II. Fickian and anomalous release from swellable devices. J. Control. Release.

[23] (2020). Test N° 437: Bovine Corneal Opacity and Permeability Test Method for Identifying i) Chemicals Inducing Serious Eye Damage and ii) Chemicals Not Requiring Classification for Eye Irritation or Serious Eye Damage.

[24] Ludwig A. (2005). The use of mucoadhesive polymers in ocular drug delivery. Adv. Drug Deliv. Rev..

[25] Meier M.M., Kanis L.A., Soldi V. (2004). Characterization and drug-permeation profiles of microporous and dense cellulose acetate membranes: Influence of plasticizer and pore forming agent. Int. J. Pharm..

[26] Di Colo G., Zambito Y. (2002). A study of release mechanisms of different ophtalmic drugs from erodible ocular inserts based on poly(ethylene oxide). Eur. J. Pharm. Biopharm..

[27] Hornyak G.L., Moore J.J., Tibbals H.F., Dutta J. (2018). Fundamentals of Nanotechnology.

[28] Pepić I., Lovrić J., Filipović-Grčić J. (2013). How do polymeric micelles cross epithelial barriers?. Eur. J. Pharm. Sci..

[29] Ghezzi M., Ferraboschi I., Delledonne A., Pescina S., Padula C., Santi P., Sissa C., Terenziani F., Nicoli S. (2022). Cyclosporine-loaded micelles for ocular delivery: Investigating the penetration mechanisms. J. Control. Release.

[30] Nguyen D.D., Luo L.J., Yang C.J., Lai J.Y. (2023). Highly Retina-Permeating and Long-Acting Resveratrol/Metformin Nanotherapeutics for Enhanced Treatment of Macular Degeneration. ACS Nano.

[31] Jacob S., Nair A.B., Shah J., Gupta S., Boddu S.H.S., Sreeharsha N., Joseph A., Shinu P., Morsy M.A. (2022). Lipid Nanoparticles as a Promising Drug Delivery Carrier for Topical Ocular Therapy—An Overview on Recent Advances. Pharmaceutics.

[32] Luo L.J., Nguyen D.D., Lai J.Y. (2021). Harnessing the tunable cavity of nanoceria for enhancing Y-27632-mediated alleviation of ocular hypertension. Theranostics.

[33] Zhang T., Jin X., Zhang N., Jiao X., Ma Y., Liu R., Liu B., Li Z. (2022). Targeted drug delivery vehicles mediated by nanocarriers and aptamers for posterior eye disease therapeutics: Barriers, recent advances and potential opportunities. Nanotechnology.

[34] Eurl Ecvam Status Report (2021). Non-Animal Methods in Science and Regulation.

[35] Efron N., Morgan P.B., Katsara S.S. (2001). Validation of grading scales for contact lens complications. Ophthal. Physiol. Opt..

