# Supplementary material for: Eudragit Films as Carriers of Lipoic Acid for Transcorneal Permeability
Source: Polymers (Basel). 2023 Apr 5;15(7):1793. doi: 10.3390/polym15071793 (PMC10097161; doi:10.3390/polym15071793)
Supplement: Supplementary file 1 [file polymers-15-01793-s001.zip › polymers-2144504-supplementary.pdf]

Nanoparticles characterization

Procedure

Nanoparticles were obtained after incubating E PO-PEG400-LA (50 mg) film in PBS buffer at 37 °C for 24 hs to ensure its complete disintegration. The supernatant was lyophilized (LABCONCO, FreeZone[18]) for one day and then analyzed by FTIR spectroscopy.

FTIR measurements

IR Spectra were recorded at ambient temperature on a SHIMADZU FTIR spectrometer using an accumulation of 40 runs in each simple in the diffuse-reflectance mode.

Figure S1 shows the resulting spectrum of EPO-PEG400-LA nanoparticles in comparison with the spectra of the pure substances. The most important bands of each substance have been assigned. We have indicated with black boxes the spectrum regions where the presence of both components in the nanoparticles is evident. Therefore, it can be seen that nanoparticles are made up of E PO and LA.

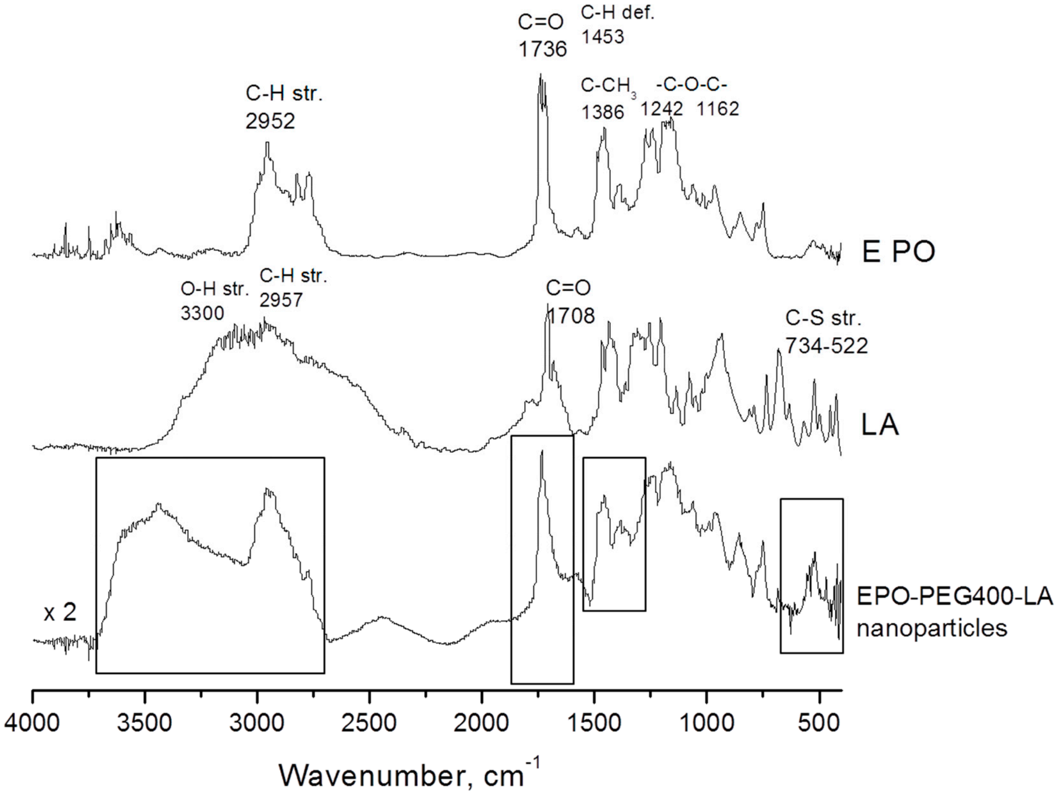

Figure S1. FITR spectra of E PO, LA and E PO-PEG400-LA nanoparticles generated from E PO-PEG400-LA (50 mg) film incubated in PBS at 37 °C for 24 hs.
